# Near-Infrared Spectroscopy-Based Frontal Lobe Neurofeedback Integrated in Virtual Reality Modulates Brain and Behavior in Highly Impulsive Adults

**DOI:** 10.3389/fnhum.2017.00425

**Published:** 2017-09-04

**Authors:** Justin Hudak, Friederike Blume, Thomas Dresler, Florian B. Haeussinger, Tobias J. Renner, Andreas J. Fallgatter, Caterina Gawrilow, Ann-Christine Ehlis

**Affiliations:** ^1^LEAD Graduate School & Research Network, University of Tübingen Tübingen, Germany; ^2^Department of Psychiatry and Psychotherapy, University Hospital Tübingen Tübingen, Germany; ^3^Department of Child and Adolescence Psychiatry, University Hospital Tübingen Tübingen, Germany; ^4^Center for Integrative Neuroscience, University of Tübingen Tübingen, Germany; ^5^Department of Psychology, University of Tübingen Tübingen, Germany; ^6^Center for Individual Development and Adaptive Education of Children at Risk, Goethe University Frankfurt Frankfurt, Germany

**Keywords:** NIRS, neurofeedback, virtual reality, impulsivity, ADHD

## Abstract

Based on neurofeedback (NF) training as a neurocognitive treatment in attention-deficit/hyperactivity disorder (ADHD), we designed a randomized, controlled functional near-infrared spectroscopy (fNIRS) NF intervention embedded in an immersive virtual reality classroom in which participants learned to control overhead lighting with their dorsolateral prefrontal brain activation. We tested the efficacy of the intervention on healthy adults displaying high impulsivity as a sub-clinical population sharing common features with ADHD. Twenty participants, 10 in an experimental and 10 in a shoulder muscle-based electromyography control group, underwent eight training sessions across 2 weeks. Training was bookended by a pre- and post-test including go/no-go, n-back, and stop-signal tasks (SST). Results indicated a significant reduction in commission errors on the no-go task with a simultaneous increase in prefrontal oxygenated hemoglobin concentration for the experimental group, but not for the control group. Furthermore, the ability of the subjects to gain control over the feedback parameter correlated strongly with the reduction in commission errors for the experimental, but not for the control group, indicating the potential importance of learning feedback control in moderating behavioral outcomes. In addition, participants of the fNIRS group showed a reduction in reaction time variability on the SST. Results indicate a clear effect of our NF intervention in reducing impulsive behavior possibly via a strengthening of frontal lobe functioning. Virtual reality additions to conventional NF may be one way to improve the ecological validity and symptom-relevance of the training situation, hence positively affecting transfer of acquired skills to real life.

## Introduction

Impulsivity refers to the inability to inhibit behavioral responses to urges created by external stimuli as well as internal desires, often brought about by the current environment. It is a ubiquitous behavioral trait found in healthy individuals as well as those with developmental disorders such as attention-deficit/hyperactivity disorder (ADHD), substance-use disorders, binge eating disorders, and others (Whiteside and Lynam, 2001; Bari and Robbins, 2013). Individual impulsive episodes, such as drunk driving, can negatively impact the lives of the impulsive individual, as well as the lives of others. On neuropsychological tasks, impulsive behavior is associated with certain types of errors, typically on conditions requiring inhibitory control. For example, the more impulsive an individual is, the more commission errors [i.e., false alarms (FA)] they make on go/no-go tasks (Aichert et al., 2012; Weidacker et al., 2016). Impulsive subgroups such as binge eaters (Hege et al., 2014) and binge drinkers (Henges and Marczinski, 2012) also make more FA than healthy controls.

From a neuroscientific perspective, impulsivity is strongly linked with dysfunctional frontal lobe activity and frontal lobe excisions (Fallgatter and Herrmann, 2001; Bari and Robbins, 2013). Development of impulse control is the result of maturation of the cognitive control network (CCN; Casey et al., 2008; Steinberg, 2008; in Shulman et al., 2016) which consists of the lateral prefrontal cortex and its connectivity with other frontal, striatal, motoric, and parietal regions (for comprehensive reviews see Cubillo et al., 2012; Rubia et al., 2013). Highly impulsive subgroups require a stronger activation of the CCN than healthy controls to achieve comparable response inhibition (Horn et al., 2003; Ding et al., 2014). Additionally, evidence for negative correlations between trait impulsiveness and activation as well as connectivity in prefrontal brain structures has been provided (Farr et al., 2012). Furthermore, there is evidence that the bilateral dorsolateral prefrontal cortex (dlPFC) may be involved in inhibitory control as transcranial direct current stimulation (tDCS) of the left dlPFC led to improved inhibitory control on a go/no-go task in participants with ADHD (Soltaninejad et al., 2015).

Neurofeedback (NF), a therapeutic technique in which participants are tasked with regulating their own brain activity, is used as a way to effect long-term change in abnormal brain activity (Arns et al., 2013). Thereby, electroencephalography (EEG)-based NF protocols have shown promise in reducing impulsive symptoms in ADHD (Gevensleben et al., 2012, 2014a). However, these protocols have had mixed effects, particularly as they are often based on brain-frequency imbalances that are highly heterogeneous within subjects (Holtmann et al., 2014). A recently emerging NF protocol for ADHD using functional near-infrared spectroscopy (fNIRS) to measure the blood oxygenation level dependent (BOLD) response within the dlPFC has several potential advantages over traditional EEG protocols (Marx et al., 2015).

Compared to EEG, fNIRS has improved spatial resolution and better correspondence of channel to underlying brain region, as well as reduced sensitivity to movement-based artifacts, making it ideal for NF training of circumscribed brain areas in motorically restless individuals (e.g., ADHD patients, children, etc.). Furthermore, evidence from BOLD-based NF paradigms suggest that they yield effects faster than their EEG-based counterparts. In a pilot study with children with ADHD, significant symptom improvements were found after only 12 sessions of fNIRS-based dlPFC training (Marx et al., 2015). Sherwood et al. (2016) found that – in healthy subjects – achieving control of the BOLD response in the dlPFC is possible after just five sessions of real-time functional magnetic resonance imaging (fMRI) NF training. Current EEG protocols, on the other hand, require between 25 and 50 sessions to realize significant effects (for a review and meta-analysis see Begemann et al., 2016). However, despite the promise of BOLD-based protocols as a potential treatment for impulsivity, such protocols still need to translate from laboratory to real-world settings.

Neurofeedback treatment is often criticized for its lack of ecological validity. Simply put, strategies of brain regulation learned in a lab setting may not translate well into the real world. Those with impulsivity struggle in the classroom where academic achievement is negatively correlated to impulsivity severity (Spinella and Miley, 2003). Therefore, any effective strategies developed in NF therapy should ultimately be applied in the classroom (or a similar real-world) setting, a concept known as transfer (e.g., Strehl, 2014). However, NF protocols – at this point – cannot be utilized in a real scholastic setting as they require large and delicate equipment, and students need to concentrate on the current lesson. An increasingly viable option, virtual reality (VR), has been used for assessment of clinical symptoms of ADHD in the classroom (Muhlberger et al., 2016) and with an EEG-based NF protocol designed to reduce inattentive and impulsive behavior in adolescents displaying behavioral problems (Cho et al., 2004). In the latter study, the VR group showed the greatest improvement following NF training on attention-related tasks relative to both a control group and a 2-D classroom group, but no difference in impulsivity. However, this study was controlled with a waiting group, thus not ruling out non-specific effects of NF training, such as continuous performance monitoring, reinforcement of compliance, and the idea that one is being treated by a sophisticated technology and professional (Gevensleben et al., 2012, 2014b). Furthermore, the NF was a separate module, not incorporated into the experience of the class itself.

Based on these findings, we developed a virtual classroom-based fNIRS NF protocol (for study design see Blume et al., 2017) in order to directly facilitate transfer of NF training effects to the classroom. Importantly, feedback is delivered in the form of gentle dimming or brightening of the overhead lighting which does not distract the participant from the experience of being in a classroom. In the present study, we implement a 2 week accelerated protocol in highly impulsive young adults, consisting of eight training sessions (one per day) which were bookended by a pre- and a post-test to assess behavioral changes during a go/no-go, n-back, and stop-signal task (SST). Changes in frontal lobe function were also assessed during the go/no-go and n-back tasks using fNIRS. To control for the previously mentioned non-specific effects of the NF training, we used bilateral musculi supraspinatus-based electromyography (EMG) biofeedback (BF) (see Marx et al., 2015; Mayer et al., 2015). This method has been successfully used in the aforementioned studies as a control for NIRS-based NF. Sham-based NF control groups (e.g., targeting putatively unrelated brain areas) invite ethical concerns, as training random areas may have unforeseeable negative effects on the participant, who is often recruited on the premise that the training will be helpful to their condition (Holtmann et al., 2014). Furthermore, participants sometimes become aware that they are part of some sham conditions (particularly if the sham feedback contains data completely unrelated to the current training situation, e.g., training data of another participant), or even assume they are part of one when they are not, leading to both drop-outs and reduced motivation, a critical aspect for any successful NF training (Birbaumer et al., 1991; Gevensleben et al., 2014a). As we did not explicitly inform participants that EMG BF was a control condition, they were less susceptible to this motivation loss.

We hypothesize that the fNIRS-based NF group will show an improvement in dlPFC activity during the cognitive tasks (go/no-go and n-back) relative to the EMG-based control group following the treatment program. We also expect the NF group to show a reduction in FA (go/no-go task) as well as reduced stop-signal reaction time (SSRT) on the SST from pre- to post-test measurement (as measures of response inhibition). As secondary outcomes, we expect reaction time (RT) and RT variability [standard deviation of the reaction time (SDRT)] to decrease for the NF group on all tasks, as the dlPFC plays a role in a multitude of executive functions. The expected neurocognitive improvements following frontal lobe focused fNIRS-based NF in a virtual training environment would confirm the general feasibility of a combination of NF with virtual training scenarios which could – in the long run – increase the ecological validity of NF interventions.

## Materials and Methods

### Participants

We recruited 22 students from the University of Tübingen out of a larger group of potential participants who had completed the Barratt Impulsiveness Scale (BIS; Barratt, 1959) using an online format. Based on their high BIS scores (*M_BIS_* = 85.75, *SD_BIS_* = 9.36), these students were selected and invited to an in-person screening for ADHD [according to criteria from the Diagnostic and Statistical Manual of Mental Disorders (DSM-IV-TR; Sass et al., 2003)] using two subtests from the Homburger ADHD Scale for Adults (HASE; Rösler et al., 2008), the German versions of the Wender Utah Rating Scale (WURS-K) and the ADHD-Self Assessment Scale (ADHS-SB). Participants meeting the criteria for an indication of ADHD under this context (WURS-K > 30 and ADHS-SB > 18) were excluded from the study and informed about the outpatient ADHD program at the Department of Psychiatry and Psychotherapy at the University Hospital Tübingen (*n* = 1). The remaining participants (*n* = 21; nine female, *M_Age_* = 23.4, *SD_Age_* = 2.8) reported no history of serious or chronic illness, neurological, or psychiatric disorders.

This study was approved by the Ethics Committee of the Medical Faculty of the University and the University Hospital of Tübingen and all procedures were in accordance with the Helsinki Declaration of 1975, as revised in 2013. Participants provided written informed consent and were compensated with 100 Euros for completing the duration of the training including pre- and post-measurements (10 sessions, 1 h each, over 2 weeks). One participant dropped out of the study due to feeling ill from the VR and was payed pro-rata of 10 Euro per hour participated.

### Study Design

The study followed a randomized, controlled experimental design. Participants were randomized (10 participants in each group) to either eight fNIRS-based NF (experimental) or eight EMG-based BF (control) sessions taking course daily over two weeks (Tuesday to Friday in the first week, Monday to Thursday in the second week). We randomized without stratifying for any other variables. Groups did not differ significantly in gender (NF: 4 female, 6 male; BF: 5 female, 5 male; Fisher’s exact test, *p* = 0.50), or in age (*M_BF_* = 22.9, *SD* = 2.88; *M_NF_* = 23.9, *SD* = 2.77; *t*(18) = 0.80, *p* = 0.44). The pre-test and post-test were exactly the same and included a go/no-go task, an n-back task, and an SST. The pre-test took place on the Monday of the first week, while the post-test occurred on the Friday of the second week. Order of the pre- and post-test measures was counter-balanced between subjects.

### Virtual Classroom Scenario

**FIGURE 1:**
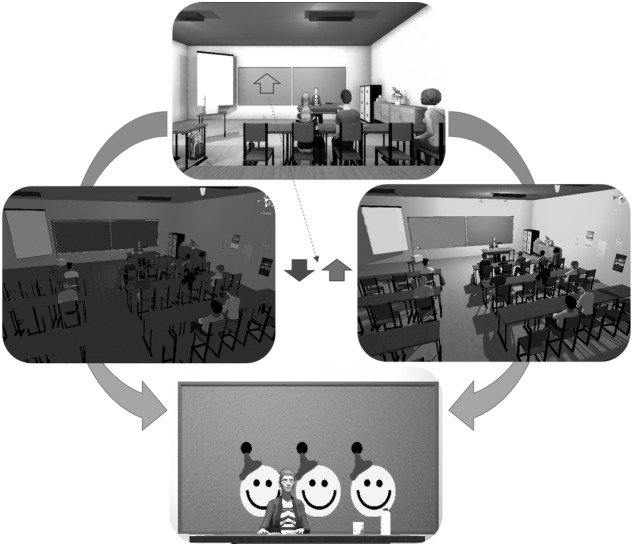
The virtual reality classroom scenario. The top image depicts the view from the participant’s head-mounted display (HMD). An arrow pointing either up or down was displayed on the chalkboard. If the arrow pointed downward, the participant should decrease the lighting in the classroom. If it pointed up, the participant should increase the lighting in the classroom. If the participant performed the task adequately, they would receive one to three smileys, presented on the chalkboard, based on the duration of success.

The participants were seated and wore the Oculus Rift (Oculus Rift, United States^1^) VR head-mounted display (HMD). The HMD rendered a virtual classroom developed by KatanaSim (KatanaSim, Germany^2^) with animated students and a teacher. The participants’ point of view was seated first-person, facing the teacher (**Figure 1**). The participant had a full 360° view from the desk seat, with other students seated nearby. The task was to control the brightness of the lighting in the classroom. When an upward-pointing arrow was shown on the chalkboard, the participant was required to “activate” in order to make the light brighter. When the arrow pointed downward, the participant was required to “deactivate” in order to make the light darker. Briefly, activation requires higher output compared to baseline from the respective feedback source, while deactivation requires reduced output compared to baseline (see below for more details on fNIRS and EMG activation/deactivation protocols). Importantly, participants were not told, in either condition, how to regulate the lighting in the classroom, they were instructed simply to try to increase the lighting in the room when the arrow pointed upward and to decrease the lighting when the arrow pointed downward. In this way, only the positive or negative feedback they received from the scenario should have enforced their learning of the feedback parameter. The probability that a trial was activation (arrow up) was 50% in sessions 1–4 and 80% in sessions 5–8. More activation was encouraged in the second half of the scenario, as more upregulation of the prefrontal cortex is associated with stronger inhibitory control (Rubia et al., 2013; Soltaninejad et al., 2015). Participants were confronted with distractions within the scenario (e.g., students turning around or cell phones ringing) from the second half of each session until the end.

Before each trial, a baseline and threshold of light fluctuation were calculated to determine the point at which the classroom light was balanced between fully bright and fully dark and the range within which it could fluctuate. Following successful activation or deactivation – when the signal was 60, 70, or 80% of the time above or below the baseline, respectively – the participant was rewarded with one, two, or three smiley faces, respectively, on the chalkboard.

Each session was comprised of three blocks, the first and the last being 12 min in length while the second, the transfer block, was 8 min. In the transfer block, the light’s brightness was fixed, meaning that the only feedback came at the end of each trial. Trial number and length varied depending on the feedback source and will be discussed in the following sections.

### fNIRS

Functional near-infrared spectroscopy records change in oxygenated (O_2_Hb) and deoxygenated (HHb) hemoglobin relative to a baseline; the amount of local O_2_Hb infers the amount of local brain activation, via the process of hemodyamic coupling, wherein increases of cortical activation lead to increases in O_2_Hb and decreases in HHb (Haeussinger et al., 2014). The ETG-4000 continuous Optical Topography System (Hitachi Medical Co., Japan) was used for pre- and post-tests as well as NF sessions.

**FIGURE 2:**
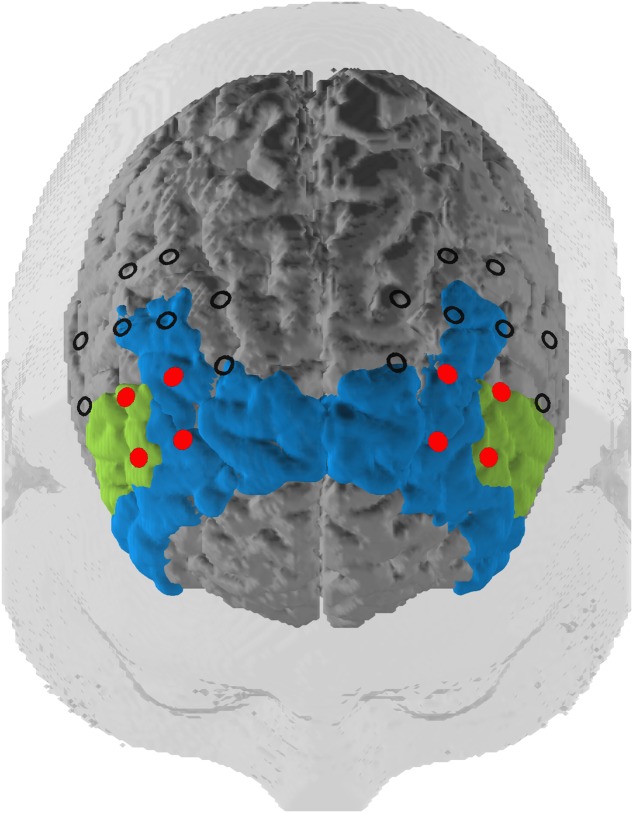
Probeset. Depiction of the target regions of the neurofeedback training (in red) and the rest of the optode array. The optodes cover a space mostly located in the dlPFC (blue, Brodmann areas 9 and 46) but also extending to the inferior frontal gyrus (green, Brodmann area 45) according to a virtual registration method (see subsection Functional Near-Infrared Spectroscopy Data).

Our optode montage featured two 3 × 3 optode arrays centered with the innermost channel of the front row of each array placed on F3 (left hemisphere) and F4 (right hemisphere) of the international 10–20 EEG system (Jasper, 1958). Source–detector distances were kept at 3 cm. The optode arrays were rotated 45° laterally along the transversal plane so that the innermost four channels in the two frontal rows were oriented over the left and right dlPFC (**Figure 2**). The third optode array was a 3 × 5 arrangement where the most superior and lateral optode on the left and right of the array were oriented on P3 and P4, respectively. Subtending the parieto-occipital cortex, this probeset was used exclusively for common average (CA) reference, a signal correction method (see below).

#### fNIRS Feedback Signal and Trials

The feedback target was the average amplitude of O_2_Hb within the bilateral dlPFC (see Marx et al., 2015). The raw fNIRS signal was sampled at 10 Hz and preprocessed in MATLAB version 9.0 (The MathWorks Inc., United States). A moving average Kalman filter with a 5 s sliding window was then applied to the data. Finally, we used a CA artifact removal method used in previous NF designs serving as a basis for this design (Marx et al., 2015; Mayer et al., 2015). This method was preferred because of its ability to remove probeset-wide effects from individual channels (Heinzel et al., 2013). For the CA, the raw average of all 46 channels was subtracted from the raw average of the eight emitter–detector channel pairings over the dlPFC in order to limit the influence of artifacts – e.g., superficial blood flow, head and jaw movements, and respiration – on the hemodynamic response in the feedback channels. All preprocessing occurred online.

**FIGURE 3:**
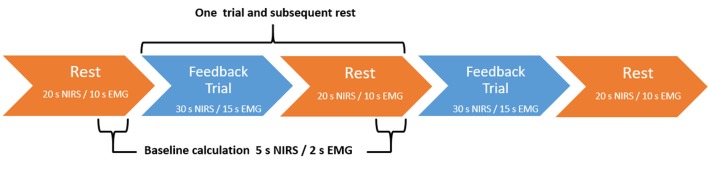
Feedback block design. fNIRS-based NF blocks consisted of either 12 NF trials and subsequent rest trials (continuous feedback blocks one and three) or 8 NF trials and subsequent rests (transfer block two). EMG-based BF blocks consisted of either 24 BF trials and subsequent rests (continuous feedback blocks one and three) or 16 BF trials and subsequent rests (transfer block two). In both conditions, blocks always began with a rest trial.

The fNIRS trials were 30 s in duration with a 5 s baseline period. Relative O_2_Hb concentration higher than baseline led to brightening of the lights; concentration lower than baseline led to dimming. Trials were divided into three blocks (**Figure 3**). The first and last blocks contained 12 trials and subsequent rests of 20 s duration. The middle block contained eight trials and rests and was used as the transfer block, wherein no continuous feedback was provided, though participants were still given feedback at the end of the trial. There was no jittering of intertrial intervals.

### EMG Feedback Signal and Trials

Monopolar EMG, with a sampling rate of 1000 Hz, provided feedback from the bilateral *musculus supraspinatus* for the control group (see Mayer et al., 2015). The signal was referenced to the right mastoid and was grounded on the left mastoid. The data stream was bandpass filtered between 80 and 300 Hz. The resulting signal was then normalized via a maximum output and a resting output, for which the participant flexed both muscles maximally for 10 s and sat completely at rest for 10 s, respectively. At each time point, feedback was equivalent to:

Feedback Index = R-L,

where R and L were the right and left normalized muscle outputs, respectively, given by:

$$\text{R}\left(\text{L}\right)\text{=}\frac{\text{Right }(\text{Left})\text{ EMG Signal-Average Resting Baseline Right }\left(\text{Left}\right)}{\text{Average Maximal Muscle Output Right }\left(\text{Left}\right)}.$$

Therefore, more tensing of right muscle led to brightening; more tensing of left muscle led to dimming. Baseline for each trial was an average of the last 2 s of the resting feedback signal.

The EMG trials were 15 s in duration with a 2 s baseline period. Relative muscular feedback index higher than baseline led to brightening of the lights; feedback index lower than baseline led to dimming. Trials were divided into three blocks (**Figure 3**). The first and last blocks contained 24 trials and subsequent rests of 10 s duration. The middle block contained 16 trials and rests and was used as the transfer block, wherein no contingent feedback was provided.

### Pre- and Post-measures

#### Go/no-go and n-Back Task

The go/no-go and the n-back tasks were programmed in Presentation version 18.0 (Neuro Behavioral Systems, United States) following previously published protocols (Mayer et al., 2015; see also Ehlis et al., 2008). We recorded fNIRS during both tasks. Briefly, the go/no-go task consisted of alternating go and no-go blocks (four repetitions each) separated by rest blocks, each block lasting 30 s. In the “go” condition, participants were asked to respond as fast as possible to each stimulus. In the “no-go” condition, participants were instructed to withhold their response on no-go trials (here: presentation of the letter “N”; 25% of trials). Dependent variables were RT, SDRT, FA, and omission errors.

The n-back task consisted of three blocks each of 2-back (high working memory load), 1-back (low working memory load), and 0-back (control) (block length: 30 s; separated by 30 s rest periods). In the 2- (1-)back task, the participants were instructed to press the space bar as quickly as possible whenever the current letter was the same as the letter two letters (one letter) back. In the 0-back task, the participant was instructed to respond when the letter “O” appeared on the screen. Dependent variables were RT, SDRT, and correct hits.

#### Stop-Signal Task

The SST followed the protocol described in Verbruggen et al. (2008). The task consisted of one practice block and three 3-min verum blocks wherein the participant should respond to the direction of an arrow pointing on the screen as quickly as possible. In roughly 25% of trials, the arrow would turn blue, indicating the participant should withhold their response, after a variable stop-signal delay (SSD) that started at 250 ms and increased or decreased by 50 ms depending on if they failed or succeeded to stop, respectively. Dependent variables in the SST included the SSRT – a measure of behavioral inhibition – RT, and SDRT. The SST was added as a secondary measure for behavior. We did not record simultaneous fNIRS with this measure.

### Analysis

#### Functional Near-Infrared Spectroscopy Data

All analysis was performed using MATLAB. In order to analyze fNIRS data, we used subroutines programmed in our research group, adapted for fNIRS from the Statistics Parametrical Mapping toolbox for MATLAB (SPM8; Friston et al., 1994). Raw signals were bandpass filtered between 0.01 and 0.2 Hz to remove unwanted physiological artifacts such as heartbeat and respiration. Next, channels exceeding three times the within-subject standard deviation over the course of the measurement were interpolated (see Hagen et al., 2014) using a Gaussian distribution with the O_2_Hb values of proximal channels given a higher weighting than distal ones; less than 10% of all channels were interpolated. We then applied a wavelet-based transform (Molavi and Dumont, 2012) to detect and correct motion artifacts that were still part of the data. We used the hmrMotionCorrectWavelet algorithm from the Homer2 fNIRS analysis package for MATLAB with the standard motion artifact detection threshold of 1.5 *SD* above the interquartile range of the data (Huppert et al., 2009). Finally, a block-related average amplitude was calculated for each channel using an interval of 0–60 s after block onset with a 10-s baseline correction. Linear detrending was applied to remove slow drifts in the data. Finally, average amplitudes over the duration of the task blocks (0–30 s) were calculated.

### Region of Interest (ROI)

We mapped fNIRS channels to corresponding, underlying cortical areas based on a virtual registration method (Rorden and Brett, 2000; Singh et al., 2005; Tsuzuki et al., 2007). The left and right dlPFC regions of interest (ROIs) consisted of the channels that we used for the NF training. These channels are concentrated in Brodmann Areas 9, 45, and 46. This includes the dlPFC and also slightly expands into the inferior frontal gyrus (IGF; see **Figure 2**).

### Rate of Learning and Correlation with Primary Outcome Variables

Additionally, we analyzed the success of the participants in obtaining control of the feedback parameter. Our success rate was calculated as the average percentage of time spent in the correct direction of the desired feedback (above or below the baseline, for activation vs. deactivation trials, respectively) for the duration of the trial. An average was calculated for all trials from the first week (four sessions) and the second week (four sessions). The rate of learning was calculated as the average of the second week minus the average of the first week. Rate of learning was then correlated with the primary outcome variables of FA rate in the no-go task and average amplitude of O_2_Hb of the feedback channels during the no-go task. Similar metrics were created in order to compute the correlations: pre–post FA errors were computed for each subject, to give a metric of individual improvement. Similarly, a post–pre average amplitude of O_2_Hb of the feedback channels was computed to reflect difference in activation after the training. In the event of significant correlations in one or more groups, we computed a pseudo-permutation test (*n* = 10,000 permutations), permuting the group assignment while keeping within-subject correlation pairs intact, to determine a significant difference between groups. The number of permutations in which the permuted group difference in ρ value was larger than the verum group difference in ρ value was divided by the total number of permutations to create a *p*-value.

### Statistical Analysis

To evaluate the statistical significance of pre–post changes in O_2_Hb and HHb in the go/no-go and n-back tasks, we conducted 2 × 2 × 2 × 2(3) repeated measures analyses of variance (ANOVAs), with the between-subjects factor treatment group (NIRS vs. EMG) and the within-subject factors of time (pre vs. post), ROI (left dlPFC vs. right dlPFC), and condition (n-back (3): 2-, 1-, and 0-back; go/no-go (2): go and no-go). For behavioral data, repeated measures ANOVAs were performed using the same factors excluding ROI. When data violated the assumption of sphericity, Greenhouse-Geisser corrected values were reported. For significant main and interaction effects, two-tailed Student’s *t*-tests were employed for *post hoc* analyses (paired or independent samples, as appropriate). In cases where the assumption of normality was violated, we used two-tailed Mann–Whitney *U* tests or Wilcoxon signed-rank tests, respectively.

### ROI Specificity

In order to determine specificity of ROIs we used pseudo-permutations tests, wherein the mean difference in the average amplitudes from pre to post measurement for a given verum ROI (vROI) for all participants was compared to a pseudo-ROI (pROI) composed of an equal number of randomly chosen NIRS channels. *N* = 10,000 permutations of pROI were calculated and the resulting *p*-value was the sum of trials in which the resulting statistic from the vROI was greater than the permuted statistic from the pROI.

## Results

### Behavioral Data

Only significant results related to the hypotheses are reported here. For a full summary of behavioral data, see **Table 1**.

**Table 1:** Behavioral data from pre- and post-test in the two experimental groups.

	Pre-test		Post-test	
		
Task	NIRS group means (±*SD*)	EMG group means (±*SD*)	NIRS group means (±*SD*)	EMG group means (±*SD*)
**Go/no-go**				
Go RT (ms)	300.0 (20.3)	289.6 (47.1)	290.5 (18.5)	293.5 (43.3)
Go SDRT (ms)	90.9 (43.1)	90.4 (39.4)	77.7 (18.1)	144.5 (99.6)
Go omission errors	0.2 (0.4)	2.9 (5.7)	0.8 (0.9)	1.6 (2.5)
No-go FA errors	4.8 (2.4)	4.8 (2.7)	2.6 (1.3)	6.0 (5.2)
No-go RT (ms)	434.6 (30.4)	417.4 (30.4)	438.3 (48.3)	411.3 (38.0)
No-go SDRT (ms)	82.0 (37.6)	81.0 (38.8)	71.1 (23.6)	79.6 (30.3)
**N-back**				
2-back Hit rate	0.95 (0.06)	0.93 (0.11)	0.98 (0.04)	0.93 (0.11)
1-back Hit rate	1 (0)	0.99 (0.03)	0.98 (0.08)	0.97 (0.04)
0-back Hit rate	1 (0)	0.99 (0.03)	1 (0)	0.96 (0.06)
2-back RT (ms)	554.2 (65.8)	485.8 (77.8)	550.3 (74.0)	433.5 (66.4)
1-back RT (ms)	473.6 (61.0)	411.7 (46.8)	491.2 (78.8)	427.2 (76.2)
0-back RT (ms)	423.9 (48.3)	388.7 (35.1)	450.0 (49.2)	418.8 (83.6)
2-back SDRT (ms)	173.1 (57.5)	163.8 (50.2)	171.7 (69.5)	87.9 (25.9)
1-back SDRT (ms)	122.6 (58.1)	71.8 (19.1)	123.5 (81.5)	82.7 (36.9)
0-back SDRT (ms)	96.6 (18.3)	139.7 (50.1)	139.7 (50.4)	113.3 (55.1)
**Stop-signal task**				
SSRT (ms)	223.5 (36.8)	224.2 (53.9)	232.2 (55.8)	223.6 (55.7)
Go trial RT (ms)	659.2 (212.8)	543.4 (96.9)	605.1 (186.5)	568.9 (102.5)
Go trial SDRT (ms)	160.8 (64.9)	125.6 (46.1)	124.1 (60.6)	145.7 (61.0)


*SD, standard deviation; RT, reaction time; SDRT, standard deviation of the reaction time; FA, false alarms; SSRT, stop-signal reaction time.*

#### Go/no-go

**FIGURE 4:**
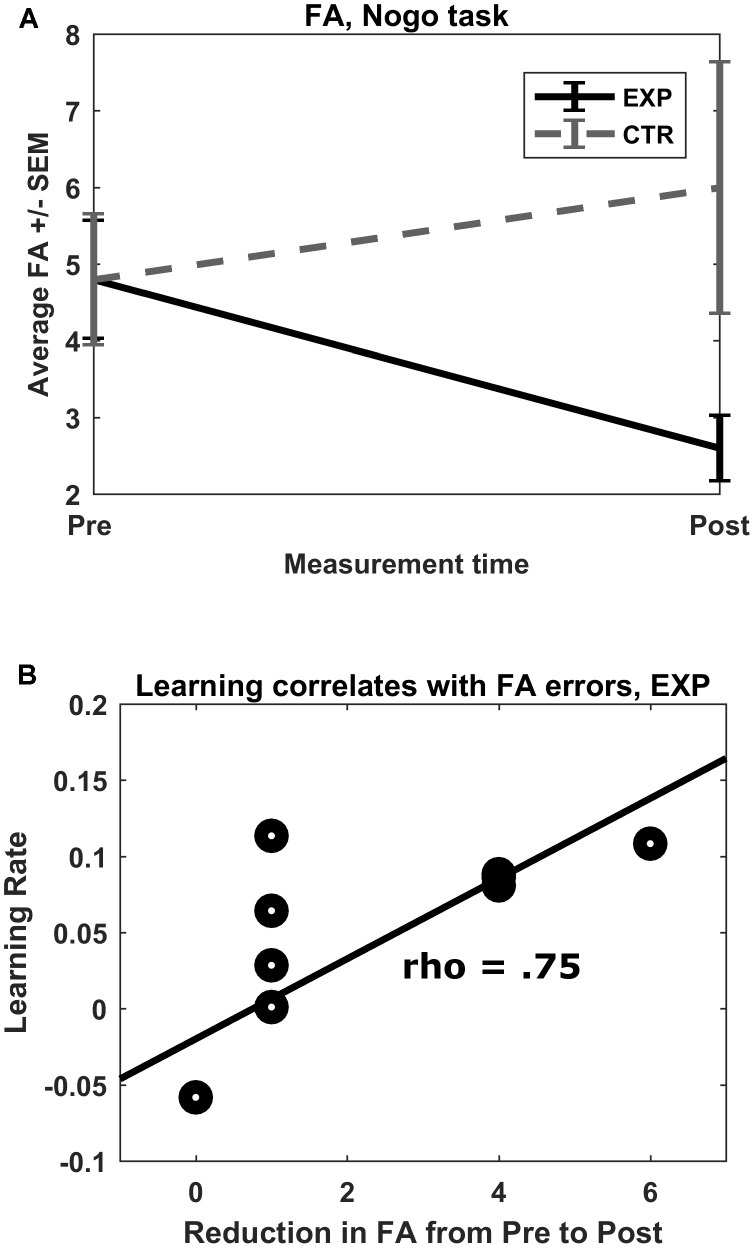
**(A)** No-go FA errors. Mean total FA errors for the no-go condition shown for both groups for both pre- and post-test. ^∗^*p* = 0.01. **(B)** Rate of learning correlation with FA reduction. In the experimental group, there was a strong correlation between rate of learning of the feedback parameter (prefrontal oxygenation) and pre–post reduction in FA errors committed.

False alarm errors in the go/no-go task showed a trend with a large effect size for a measurement time^∗^group interaction effect (*F*(1,18) = 4.08, *p* = 0.059, η^2^ = 0.185). *Post hoc* Wilcoxon signed-rank tests revealed a reduction of FA errors from pre to post measurement in the experimental group (*M_pre_* = 4.8, *SD_pre_* = 2.4; *M_post_* = 2.6, *SD_post_* = 1.3; *Z* = -2.57, *p* = 0.01), but not in the control group (*M_pre_* = 4.8, *SD_pre_* = 2.7; *M_post_* = 6.0, *SD_post_* = 5.2; *Z* = -0.30, *p* = 0.77) (**Figure 4A**). No other interaction effects were observed.

#### Rate of Learning

A one-sample Kolmogorov–Smirnov test rejected the null hypothesis that the learning rates for the first half and second half of the experimental and control groups, respectively, followed a normal distribution (*D* = 0.65, 0.65, 0.64, 0.65, *N* = 10 each, and *p* < 0.05 each). Therefore, Wilcoxon signed-rank tests and Spearman correlations were calculated. For the experimental group, there was no significant difference between first half and second half performance, but a medium effect size indicating better second half performance (*Z* = 1.48, *p* = 0.13, *r* = 0.33). There was, however, a significant difference between first and second half performance for the control group (*Z* = 2.68, *p* = 0.013, *r* = 0.60), indicating a significantly better performance in the second week with a large effect size.

The rate of learning of both groups failed to correlate significantly with post–pre changes in average O_2_Hb concentration in feedback channels (|ρ| < 0.224, *p* > 0.05). The rate of learning in the experimental group, however, correlated strongly with size of pre–post reduction in FA (ρ = 0.75, *p* = 0.013; see **Figure 4B**). Rate of learning in the control group did not correlate with pre–post reduction in FA (ρ = -0.24, *p* = 0.508). The resulting pseudo-permutation test concluded that there was a significant group difference (*p* = 0.015).

#### N-Back Task

No significant behavioral interaction effects were observed. Hit rates for each condition were nearly 100% in the pre-test. Furthermore, no FA errors were made in this task. A ceiling effect was evident for this task.

#### Stop-Signal Task

Reaction time variability yielded a significant interaction effect of measurement time^∗^group (*F*(1,18) = 5.39, *p* = 0.03, η^2^ = 0.231), with the experimental group showing significantly reduced RT variability following the training (*M_pre_* = 160.78 ms, *SD_pre_* = 64.88; *M_post_* = 124.13, *SD_post_* = 60.60; *t*(9) = 2.48, *p* = 0.035). The control group showed no difference between measurements (*M_pre_* = 125.55 ms, *SD_pre_* = 46.13; *M_post_* = 145.70, *SD_post_* = 61.04; *t*(9) = 1.04, *p* = 0.328).

### fNIRS Data

#### Go/no-go O_2_Hb

**FIGURE 5:**
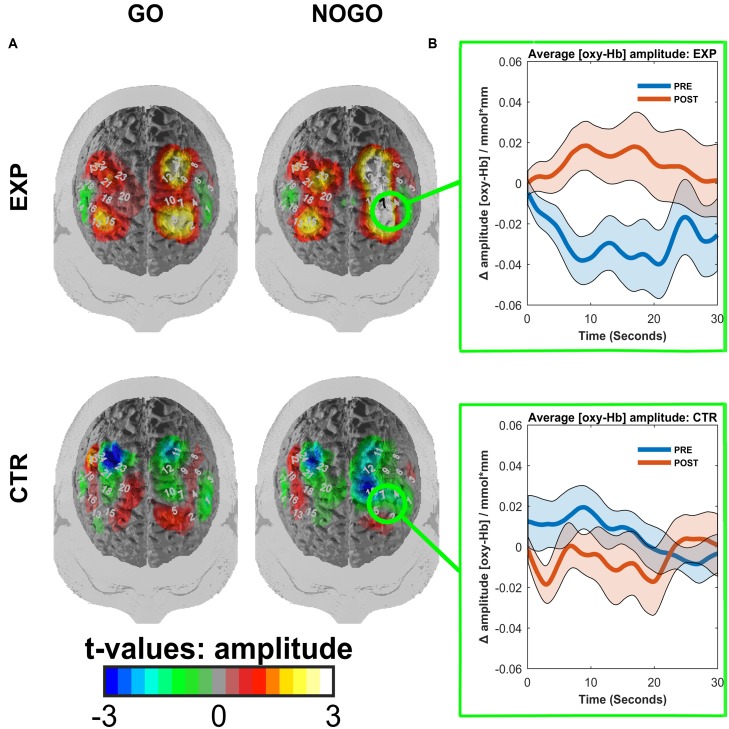
**(A)** Contrasted t-maps of the average amplitudes of O_2_Hb for different blocks of the go/no-go task. Contrasts represent post-test values minus pre-test values. CTR represents the EMG control group, while EXP represents the experimental fNIRS group. *T*-values were obtained by *t*-tests corrected for multiple comparisons with the Armitage–Parmar correction. Positive channels indicate stronger activation in the post-relative to the pre-test. Significant channels are depicted in black. **(B)** ROI event-related averages. Circled regions from **(A)** indicate the left dlPFC ROI for which the event-related average of O_2_Hb ± standard error of the mean (SEM) is depicted for both pre-(blue) and post-(red) tests.

We observed a main effect of task (*F*(1,18) = 11.92, *p* = 0.003, η^2^ = 0.398, mean amplitudes: *M_go_* = 0.005, *SD* = 0.033 mm^∗^mol/l, *M_no-go_* = -0.005, *SD* = 0.029 mm^∗^mol/l) and an interaction effect of time^∗^task^∗^ROI^∗^group (*F*(1,18) = 5.63, *p* = 0.029, η^2^ = 0.238). This interaction was caused by a pre to post increase in O_2_Hb amplitudes of the left dlPFC in the experimental group during the no-go task (*M_pre_* = -0.029, *SD* = 0.035 mm^∗^mol/l; *M_post_* = 0.010, *SD* = 0.040 mm^∗^mol/l; *t*(9) = -3.63, *p* = 0.005; see **Figure 5**). In the control group of the same condition, time, and ROI, there was no significant change (*M_pre_* = 0.006, *SD* = 0.017 mm^∗^mol/l; *M_post_* = -0.006, *SD* = 0.031 mm^∗^mol/l; *t*(9) = 1.15, *p* = 0.281). All other *post hoc* comparisons failed to reach significance (|*t*(9)| < 1.837, *p* > 0.1). The permutation test indicated that this ROI was indeed the focal point for the increase in brain activation. The resulting *p*-value was equal to *p* = 0.003, indicating that there is high spatial specificity to the activation, located in the left dlPFC.

#### Go/no-go HHb

We observed no main effects, only an interaction effect of task^∗^hemisphere (*F*(1,18) = 5.79, *p* = 0.027, η^2^ = 0.243). *Post hoc* testing indicated that there was a trend toward a significant difference in HHb activation between the left and right hemisphere in the “go” condition (*M_left_* = -0.005, *SD* = 0.023; *M_right_* = -0.013, *SD* = 0.026; *t*(9) = 2.07, *p* = 0.052).

#### N-Back O_2_Hb

We observed no main effects or significant interaction effects (all |*F*(2,36)| < 2.50; all *p* > 0.11).

#### N-Back HHb

We observed a trend for a main effect of task (*F*(1.39,24.93) = 3.75, *p* = 0.052, η^2^ = 0.173; mean amplitudes: *M*_2*Back*_ = -0.011, *SD* = 0.023; *M*_1*Back*_ = -0.007, *SD* = 0.021; *M*_0*Back*_ = 0.001, *SD* = 0.024). Again, the indication is a higher activation in tasks with a higher working load. We also observed a trend for a main effect of time (*F*(1,18) = 3.26, *p* = 0.088, η^2^ = 0.153; *M_pre_* = -0.008, *SD* = 0.026; *M_post_* = -0.003, *SD* = 0.021), indicating a marginal decrease in activation across all tasks from pre to post measurement time. No other main effects or interaction effects were observed.

## Discussion

The present study was designed to test the efficacy of a novel neurofeedback intervention (fNIRS-based frontal lobe NF in a virtual classroom environment) with the ultimate aim of reducing ADHD symptoms in schoolchildren by increasing their ability to regulate prefrontal cortex activity (Blume et al., 2017). Here, we focused on the effects of this newly developed NF protocol in a sample of highly impulsive young adults, a sub-clinical risk population that exhibits many of the behavioral abnormalities also seen in patients with ADHD (e.g., Herrmann et al., 2009). In this proof-of-concept study, we were primarily interested in first, whether the fNIRS-based NF group would show increased cortical activation in feedback channels during frontal lobe/impulsivity-related tasks (go/no-go and n-back), following focused training of these channels and second, whether the fNIRS-based NF group would show a reduction in impulsive behaviors (go/no-go, n-back, SST).

During a go/no-go task, we observed a significant increase compared to a pre-training baseline in cortical O_2_Hb concentration in the left dlPFC of the experimental (fNIRS) group only. During the same task, we observed a concurrent and significant reduction in FA errors of the same group. Importantly, this reduction in FA errors correlated significantly with the rate of learning of the experimental subjects but not the control subjects. Additionally, we observed a reduction in RT variability on the SST for the experimental group. We observed no group differences in either cortical activation or behavior on the n-back task. The lack of a group difference after training on this task is likely due to the study specifically focusing on the recruitment of highly impulsive students. There is no evidence to suggest that highly impulsive participants have explicit deficits in working memory. In fact, in a study examining the correlations between trait impulsivity (as measured by BIS self-report) and performance on various neurocognitive tasks, no significant correlation was found between trait impulsivity and working memory performance, while trait impulsivity correlated strongest with go/no-go errors (Keilp et al., 2005). Furthermore, task accuracy reflected a ceiling effect from the pre-test, indicating that the task was not difficult for these subjects. Therefore, despite the potential benefit to working memory that training the dlPFC might imbue, in our case there may have been no deficit to correct. Lastly, HHb data showed no differences in activation in either task. These results make sense in the context of the NF training; since O_2_Hb was trained, the hypothesis would be that O_2_Hb and not HHb would show the strongest pre–post effects. In addition, O_2_Hb is more sensitive to detection of changes than HHb (Strangman et al., 2002).

False alarm errors, or incorrect go-responses to no-go stimuli, represent a failure to exhibit response inhibition (Aichert et al., 2012), an impulsive trait that subjects with ADHD share with highly impulsive participants. A reduction for the experimental group and not for the control group suggests that the fNIRS intervention was effective in reducing impulsive behavior as specified. The strong O_2_Hb correlation observed between a reduction in FA errors and the rate of learning within the experimental group, but not within the control group, further illustrates the importance of specificity in NF training. The goal of actually learning to control the feedback parameter is often overlooked in NF studies, where the rate of obtained control is rarely reported (Zuberer et al., 2015). Interestingly, the control group showed a significant improvement between the first and second week in regulating the feedback parameter while the experimental group did not. This likely has to do with the comparable ease of the EMG feedback; once one learns the correct movement, it can relatively easily be replicated every trial. The fNIRS feedback is likely more complex, as there is no right or wrong way to achieve the feedback parameter, and sustaining oxygenation of the dlPFC over time is strenuous. Given this complexity, the medium effect size observed in the fNIRS learning rate is encouraging, and may simply mean that more sessions are needed to fully gain control. Moreover, for the specific sample investigated and trained here (i.e., highly impulsive subjects), frontal lobe alterations have been shown as a central neurophysiological correlate, so it is perhaps not surprising that improving control over this area of the brain seems to have been particularly difficult. However, this behavioral effort seems to pay dividends, as we see that the more control impulsive subjects were able to gain over the activation of their dlPFC, the fewer FA errors they made, whereas the successful learning of the EMG parameter had little effect. This result supports the findings of an fNIRS study that sought to differentiate the roles of the medial and lateral prefrontal cortex during a go/no-go task. The bilateral middle frontal gyrus (i.e., the dlPFC) was responsible for error monitoring during the motor inhibition segment of the go/no-go task (Rodrigo et al., 2014). Our results indicate that the combination of both correct feedback parameter (i.e., frontal lobe focused) and successful learning of that parameter, not one or the other in isolation, is important to the feedback’s overall success.

The task-specific increase in prefrontal oxygenation coinciding with a reduction in FA errors suggests that – following the frontal fNIRS training – the highly impulsive participants were able to recruit more cognitive resources, particularly from the dlPFC, during this task, leading to improved performance. Whether or not this was intentional is a matter of debate, but the goal of NF interventions remains to train implicit activation of brain activity through operant and classical conditioning (Strehl, 2014). Therefore, it seems that the participants were able to transfer skills learned either implicitly or explicitly from the training into a performance situation. Furthermore, this increase in cortical activation was both task- (no-go) and region-specific (left dlPFC). While there was no increase in activation in the right dlPFC, the left-specific increase as well as the increase in inhibitory control are in line with the tDCS study of Soltaninejad et al. (2015) who used cathodal stimulation over the left dlPFC of adolescents with ADHD and observed a decrease in FA errors. While the literary consensus places the locus of inhibitory control within the right dlPFC, inferior prefrontal, premotoric, and striatal brain structures (Aron et al., 2004, 2014; Bari and Robbins, 2013; Obeso et al., 2013), the left dlPFC shares strong functional connectivity with the above-mentioned areas (Ridderinkhof et al., 2004; Aron et al., 2014). Moreover, the dlPFC does not seem to be directly responsible for inhibitory control, but rather functions as a higher order mechanism that organizes the relevant brain structures above when attention control or increased working memory capacity is needed, in particular for oddball or complex no-go tasks (Criaud and Boulinguez, 2013). Because our go/no-go paradigm could be considered oddball, with an occurrence of no-go stimuli in only 25% of trials, it may be that the extra dlPFC resources recruited were used for focusing attention, rather than inhibitory control *per se*. Indeed, the reduction in SDRT seen in the SST also indicates an increase in attentional resources, possibly also mediated by an increase in prefrontal brain activity, though NIRS data were not available for this task. Increases in SDRT are generally considered to be related to lapses in attention (Alderson et al., 2007), though Kirkeby and Robinson (2005) found SDRT to be inversely correlated with trait impulsivity. Still, this does not rule out the idea that our impulsive sample also suffered from inattentiveness.

Treatment effects for both impulsivity and possibly inattention are encouraging from a translational perspective regarding potential use of our NF design with an ADHD population. We chose the dlPFC as a NF site because of its involvement in general top-down cognitive control, and the realization of significant training effects in impulsivity and possible inattention suggests that the protocol may be useful for an ADHD population. Several reasons lead us to be hopeful of even greater effects in a current study in our lab with ADHD schoolchildren (Blume et al., 2017). First, the sample size of this study was small. Only large effects could be detected, and with a greater sample size, we would expect to see effects in a wide range of other cognitive and behavioral deficits. Secondly, the training was compact and about half the number of training sessions we would recommend (and currently use) for a clinical ADHD project. As far as we know, this is the shortest number of training sessions to produce effects in brain activation and behavior that was adequately controlled for specificity. Cho et al. (2004) also used a 2 week, eight session NF paradigm with EEG and found training effects for inattention and impulsivity, but they did not have an adequate control group (waiting group), and additionally, did not measure differences in brain activity pre and post. Lastly, but most importantly, children have a greater capacity for brain plasticity than adults (Kolb and Gibb, 2011). For children with ADHD, this capacity is even more pronounced within the dlPFC, a region that develops particularly late for them (Rubia et al., 2013). Given the current study’s results, we would expect even greater improvements within a child population.

The current study was limited by several factors, which we hope to improve upon in a second study with children with an ADHD diagnosis (Blume et al., 2017). The sample size was small which limited data analysis. Our aim was to test the viability of an immersive VR NF paradigm, and it appears that the full classroom immersion did not detract from the ability of the participants to regulate their brain activity. There was a difference between experimental groups in pre-test no-go activation, with the experimental group showing less activation than the control group. Small groups, even with proper randomization, have a much greater chance of having differing baseline measurements simply due to sampling error (Marshall, 1996). The larger the group, the smaller the chance of pre-baseline differences due to a random sampling error. As NF studies require large time and monetary investments per participant, and the aim of our study was to ultimately test the efficacy of VR NF, we chose 10 participants per group as a balance between power and realism. For technical reasons, we did not have triggers to compare the extent to which participants were able to regulate their brain activity across sessions, something that will be improved in the next study. While we used distractors in the current study, there was no way to compare trials in which a distractor occurred to trials in which they did not. Furthermore, we lack a comparison of the effects of the immersive VR NF paradigm to a 2-D version. In an ongoing study with children with ADHD (Blume et al., 2017), we include a 2-D group that still uses lighting in the classroom as the feedback source, but the child sees the classroom on a normal computer monitor. In this way, we will be able to determine if immersive NF is actually more effective for the transfer of the learned regulation. Furthermore, the classroom itself is only one of many possible VR NF designs. Virtual reality scenarios coupled with NF are limited only to the imagination and relevance to a certain psychological disorder. Virtual reality NF with subjects with social phobias, for example, could be integrated within a potentially stressful social situation, like a bar or dinner party, furthering the ecological validity of the treatment while also avoiding an exposition-driven therapeutic approach that cannot be as easily controlled.

Considering these limitations and the relative ease with which they could be improved upon going forward, it seems that VR NF is a very promising modality for the treatment of behavioral disorders with known pathophysiological alterations.

## Ethics Statement

This study was approved by the Ethics Committee of the Medical Faculty of the University and the University Hospital of Tübingen and all procedures were in accordance with the Helsinki Declaration of 1975, as revised in 2013.

## Author Contributions

All authors have approved of the final version of this manuscript. JH study design, data collection and analysis, and manuscript preparation; FB study design, data collection, and manuscript preparation; TD study design and manuscript preparation; FH data analysis; TR study design and manuscript preparation; AF study design and manuscript preparation; CG study design and manuscript preparation; and A-CE study design, data analysis, and manuscript preparation.

## Conflict of Interest Statement

The authors declare that the research was conducted in the absence of any commercial or financial relationships that could be construed as a potential conflict of interest.

## Footnotes

1 www.oculusvr.com

2 www.katanasim.com
